# Predictors of Early Neurological Improvement in Patients with Anterior Large Vessel Occlusion and Successful Reperfusion Following Endovascular Thrombectomy—Does CT Perfusion Imaging Matter?

**DOI:** 10.1007/s00062-022-01147-0

**Published:** 2022-03-04

**Authors:** Yan Li, Natalie van Landeghem, Aydin Demircioglu, Martin Köhrmann, Elias Kellner, Lennart Milles, Benjamin Stolte, Andreas Totzeck, Philipp Dammann, Karsten Wrede, Jens Matthias Theysohn, Hanna Styczen, Michael Forsting, Isabel Wanke, Benedikt Frank, Cornelius Deuschl

**Affiliations:** 1 Institute of Diagnostic and Interventional Radiology and Neuroradiology, University Hospital Essen, University of Duisburg-Essen, Hufelandstr. 55, 45147 Essen, Germany; 2 Department of Neurology and Center for Translational Neuro- and Behavioral Sciences (C-TNBS), University Hospital Essen, Hufelandstraße 55, 45147 Essen, Germany; 3 Medical Physics, Department of Radiology, University Medical Center Freiburg, Killianstraße 5a, 79106 Freiburg, Germany; 4 Department of Neurosurgery and Spine Surgery, University Hospital Essen, Hufelandstraße 55, 45147 Essen, Germany; 5 Swiss Neuroradiology Institute, Bürglistraße 29, 8002 Zurich, Switzerland

**Keywords:** Stroke, Thrombectomy, Endovascular thrombectomy, CT perfusion, ASPECTS

## Abstract

**Purpose:**

We aimed to investigate treatment effect of endovascular thrombectomy (EVT) on the change of National Institutes of Health Stroke Scale (NIHSS) scores in acute ischemic stroke (AIS) patients with anterior large vessel occlusion (LVO). Predictors of early neurological improvement (ENI) were assessed in those with successful reperfusion.

**Methods:**

Data on stroke patients from January 2018 to December 2020 were retrospectively analyzed. Anterior LVO was defined as occlusion of internal carotid artery and/or M1/M2 branch of middle cerebral artery. A reduction of at least 8 NIHSS points at 24 h after EVT or NIHSS score ≤ 1 at discharge was defined as ENI. In patients with successful reperfusion (TICI score of 2b/3) and available CT perfusion (CTP) imaging, 20 variables were tested in a smoothed ridge regression for their association with ENI.

**Results:**

One hundred seventy two out of 211 patients had successful perfusion with 54 patients achieving ENI. Impact of successful EVT on reducing NIHSS score grew continuously on a daily basis up to the date of discharge. 105 out of 172 patients were included in final regression model. Short time from onset to admission and from groin-puncture to reperfusion, young age, low prestroke disability, high baseline CTP ASPECTS and high follow-up non-contrast CT (NCCT) ASPECTS were significantly associated with ENI. Neither baseline NCCT ASPECTS nor the volume of penumbra or ischemic core measured on CTP were associated with ENI.

**Conclusion:**

CTP ASPECTS might better predict ENI than non-contrast CT at baseline in patients with successful reperfusion following EVT.

## Introduction

Since 2015 multiple randomized trials showed efficacy of endovascular thrombectomy (EVT) over standard medical treatment alone regarding improvement of functional outcomes in patients with acute ischemic stroke (AIS) caused by anterior large vessel occlusion (LVO) within 7.3 h from stroke onset to arterial access [1, 2]. In selected patients with evidence of salvageable brain tissue on CT or MR perfusion imaging, the time window for EVT might be extended to 24 h after patients were last seen to be well because of higher percentage of functional independence, lower 90-day mortality rate, and absence of considerably increased risk of brain hemorrhage compared to standard medical treatment alone [3, 4]. After translating these findings into clinical practice, the question arose whether EVT is beneficial for all patients and which prognostic factors are important for a favorable neurological outcome in addition to endovascular reperfusion. In the setting of stroke triage, assessment of those determinants in a given patient might not only assist rapid decision-making but also help to optimize postthrombectomy care concerning distribution of patients in stroke unit or intensive care unit.

Hence, we conducted a retrospective analysis to study the treatment effect of EVT on the change of National Institutes of Health Stroke Scale scores (NIHSS) scores at 24‑h intervals from admission to discharge in patients with anterior LVO. Moreover, we aimed to assess predictors associated with favorable early neurological improvement (ENI) in patients with successful reperfusion following EVT. Particular attention was given to cerebral CT perfusion (CTP) imaging, which enables a more reliable assessment of ischemic change, as non-contrast CT (NCCT) often shows no or only subtle infarct demarcation at baseline, especially in patients with an early time window < 3 h [5, 6]. Based on our own observation, we hypothesized that CTP-derived parameters at baseline might be more predictive of clinical outcome than NCCT.

## Material and Methods

### Study Population

**Fig. 1:**
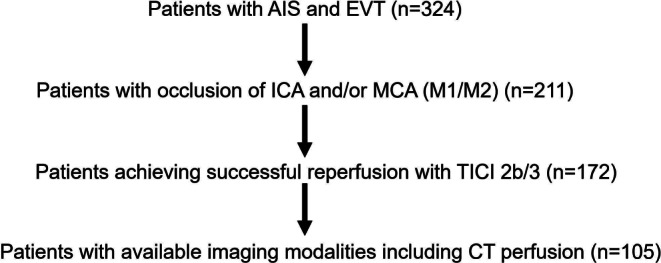
Flow chart of selection process with numbers of patients. *AIS* acute ischemic stroke, *ICA* internal carotid artery, *MCA* middle cerebral artery, *TICI* thrombolysis in cerebral infarction scale, *CT* computed tomography, *EVT* endovascular thrombectomy

In this single-center retrospective analysis, we reviewed all EVT cases from January 2018 to December 2020 at our tertiary care center in patients with AIS due to anterior LVO. First, the impact of EVT on the dynamic change in NIHSS score was evaluated. The inclusion criteria were as follows: (1) occlusion of internal carotid artery and/or M1/M2 of middle cerebral artery, (2) available NCCT at admission and 24 h after EVT with calculated Alberta Stroke Program Early CT Score (ASPECTS). Then we performed subgroup analysis to test the association of each variable with ENI in patients with successful reperfusion defined by the thrombolysis in cerebral infarction scale (TICI) of 2b/3 [7] and additionally available CT perfusion imaging at baseline (see flow chart Fig. 1). CT perfusion imaging belongs to standard stroke imaging protocol for all incoming stroke patients brought primarily by emergency ambulance. A small part of patients were secondarily transferred to our angio suite for endovascular treatment from peripheral primary care centers, which usually did not perform CT perfusion imaging.

## Clinical and Neurological Assessment

Patients were categorized into groups with witnessed and unwitnessed onset of symptoms. In the case of unwitnessed onset of symptoms, the time the patient was last known well was defined as symptom onset. Time points of symptom onset, hospital admission, groin puncture and final reperfusion at the end of EVT were documented. At admission, baseline patient characteristics such as age, gender, history of diabetes mellitus (DM), coronary artery disease (CAD) and presence of atrial fibrillation were assessed. The NIHSS was used to quantify neurological impairment at admission, 24/48/72 h after EVT and at discharge. Additionally, the degrees of prestroke disability were classified with modified Rankin Scale (mRS).

### Neuroimaging Analysis

At admission, extent of cerebral early ischemic change was evaluated by using ASPECTS on baseline NCCT, while the site of vessel occlusion was determined on CT angiography. The volume of hypoperfused tissue was estimated in areas with a Tmax exceeding 6 s, and the volume of ischemic core was calculated for areas with cerebral blood flow (CBF) less than 30% compared to the unaffected hemisphere on CTP imaging provided by automatic postprocessing software VEOcore (VEObrain GmbH, Freiburg, Germany) [3]. The volume of penumbra was calculated as volume difference of hypoperfused tissue and ischemic core. We used a 10-point quantitative topographic scale to calculate the CTP ASPECTS [8]. Similar to ASPECTS on NCCT, 1 point is subtracted from the initial 10 points for presence of ischemic core (areas with CBF less than 30% compared to the unaffected hemisphere) in each of the predefined regions in ASPECTS.

Technique of EVT was performed based on the decision of the neurointerventionalist. Combination of stent-retriever and contact aspiration was the primary thrombectomy technique, while aspiration alone was sometimes preferred for the first pass in cases of distal M1 occlusion. Depending on the final angiographic result, the grade of reperfusion was determined with TICI score. The first pass effect, defined as TICI 3 after first pass, was documented. Follow-up NCCT was performed after 24 h for reassessment of ischemic change with ASPECTS and for detection of bleeding events classified by Heidelberg bleeding classification [9]. Symptomatic intracranial hemorrhage [10], defined as a deterioration of at least 4 points in the NIHSS score associated with brain hemorrhage or hemorrhage leading to death, was documented.

Two neuroradiologists (YL and CD, both with > 5 years of experiences), blinded to the patient’s clinical and neurological information, independently reviewed all neuroimaging modalities and calculated the scores subsequently. Discrepancies were resolved by consensus meeting.

### Primary Neurological Outcome

As suggested in previous trials, ENI defined as a dramatic decrease of NIHSS scores at 24 h after EVT represents an accurate and valid outcome measure of treatment effect and correlates highly with mRS at 90 days [6–8]. Further, it was also proposed as a primary outcome for clinical trials [11]. Accordingly, ENI was set as primary neurological outcome in our study. A decrease of 8 or more points at 24 h or an NIHSS score of less than 2 at discharge was defined as ENI [12, 13].

### Statistical Analysis

For descriptive analysis, numerical variables were expressed as means with 25–75% interquartile ranges (IQR) and categorical variables were presented as frequencies and percentages. Patients were categorized in ENI+ and ENI− groups, between which the baseline characteristics, clinical, neurological and neuroimaging parameters were compared with each other using χ^2^-test for nominal and Mann-Whitney U test for numerical variables. Correlations between variables and the change in NIHSS score at 24 h were calculated by using Spearman’s rank-order correlation test.

Taking ENI as dependent variable, a smoothed ridged regression model with a logit link was performed to estimate the predictive value of each variable. The predictors included age, sex, presence of DM, CAD and atrial fibrillation, NIHSS score at admission, mRS before stroke, witnessed/unwitnessed stroke, baseline ASPECTS and CTP ASPECTS, volume of ischemic core, volume of penumbra, use of intravenous thrombolysis, time intervals from symptom onset to admission, door to groin, groin puncture to reperfusion, first pass effect of EVT, follow-up ASPECTS on NCCT and brain hemorrhage on follow-up NCCT at 24 h as well as symptomatic hemorrhage. The smoothed ridge regression model was preferred over the more standard logistic regression because of the large number of covariates [14].

All descriptive statistics were performed with SPSS (version 27, IBM, Armonk, NY, USA). The smoothed ridge regression was computed using the R‑Software environment for statistical computing (version 4.3, R Foundation for Statistical Computing, Vienna, Austria) and the islasso library. A *p*-value < 0.05 was considered statistically significant.

## Results

### Baseline Characteristics and Neurological Outcome

We identified a total of 324 patients that underwent EVT and 211 patients (122 females) presented with anterior LVO. Witnessed strokes were ascertained in 114 (54%) patients. The mean age was 76 years (IQR 66–82 years). Atrial fibrillation was present in 112 (53.1%), DM in 55 (26.1%) and CAD in 54 (25.6%) patients. Most of the patients were functionally independent in the daily activities before stroke onset (mean value of mRS 1, 80% with mRS ≤ 2). The mean NIHSS scores was 15 (IQR 9–20) at hospital admission. The mean baseline ASPECTS and CTP ASPECTS were 10 (IQR 9–10) and 9 (7–10), respectively, and 133 (63%) patients received intravenous thrombolysis. Successful reperfusion represented 81.5% (TICI 2b: 30.3%; TICI 3: 51.2%) of all cases with 54 cases of first pass effect (24.6%). The mean NIHSS scores 24/48/72 h after EVT and at discharge were 13.5 (IQR 5–26), 12 (5–24), 10 (4.25–21) and 8 (3–42), respectively. ENI was found in 59 patients (28% of 211). The mean follow-up ASPECTS 24 h after EVT was 8 (IQR 6–10). Brain hemorrhage was detected in 41 (19.4%) patients and symptomatic intracranial hemorrhage (SICH) in 19 (9%) patients. The baseline characteristics and outcomes are summarized in Table 1.

**Table 1:** Baseline characteristics, endovascular and neurological outcomes of all patients

Variables	All (*n* = 211)	ENI+ (*n* = 59)	ENI− (*n* = 152)	*p*-Values
Age (years)	76 (66–82)	73 (62–80)	77 (61.25–83)	0.054
Male sex	89 (42.4%)	24 (40.7%)	65 (42.8%)	0.877
DM	55 (26.1%)	13 (22%)	42 (27.6%)	0.486
CAD	54 (25.6%)	13 (22%)	41 (27%)	0.489
Atrial fibrillation	112 (53.1%)	27 (45.8%)	85 (55.9%)	0.219
Witnessed stroke	114 (54%)	34 (57.6%)	80 (52.6%)	0.541
Symptom onset to hospital admission (min)	170 (58–482)	145 (50.25–405.5)	177.5 (62.25–516.75)	0.172
Door-to-groin (min)	66 (42–84)	62 (31–86)	66 (48–83)	0.486
Groin puncture to reperfusion (min)	73 (44–108)	64 (40–88)	82.5 (47.75–122.74)	*0.002*
mRS before stroke	1 (0–2)	0 (0–2)	1 (0–2)	*0.012*
NIHSS at admission	15 (9–20)	14 (10–19)	15 (9–20.75)	0.868
NIHSS at 24 h	13.5 (5–26)	4 (2–8)	19 (11–32)	*<* *0.0001*
NIHSS at 48 h	12 (5–24)	3.5 (1–6.25)	17 (10–27)	*<* *0.0001*
NIHSS at 72 h	10 (4.25–21)	3 (1–6)	15 (8–26.5)	*<* *0.0001*
NIHSS at discharge	8 (3–42)	2 (1–4)	13.5 (6–42)	*<* *0.0001*
Baseline ASPECTS	10 (9–10)	10 (9–10)	10 (9–10)	0.328
Baseline CTP ASPECTS	9 (7–10)	9 (7–10)	9 (7–10)	0.754
Volume of penumbra (ml)	69.5 (43.25–115.75)	66 (40–111)	76 (44–118)	0.397
Volume of ischemic core (ml)	17 (2.25–44.5)	14 (5–45)	17 (0–44)	0.812
Intravenous thrombolysis	133 (63%)	40 (67.8%)	93 (61.2)	0.428
TICI 2b/3	172 (81.5%)	54 (91.5%)	118 (77.6%)	*0.019*
First pass effect	52 (24.6%)	21 (35.6%)	31 (20.4%)	*0.016*
Follow-up ASPECTS	8 (6–10)	9 (8–10)	8 (5–9.25)	*<* *0.0001*
Hemorrhage on follow-up CT	41 (19.4%)	4 (6.8%)	37 (24.3%)	*0.003*
SICH	19 (9%)	1 (1.7%)	18 (11.8%)	*0.029*

Numerical variables were expressed as means with 25–75% interquartile ranges (IQR). χ^2^-test for nominal and Mann-Whitney U test for numerical variables were used for comparison between patient groups with and without ENI. Significant *p*-values are marked in *italics*

*ENI* early neurological improvement, *DM* diabetes mellitus, *CAD* coronary artery disease, *mRS* modified Rankin scale, *NIHSS* National Institutes of Health Stroke Scale, *ASPECTS* Alberta Stroke Program early CT Score, *CTP* CT perfusion, *TICI* thrombolysis in cerebral infarction scale, *SICH* symptomatic intracranial hemorrhage

## Treatment Effect of EVT on ENI and Change of NIHSS Scores

**Fig. 2:**
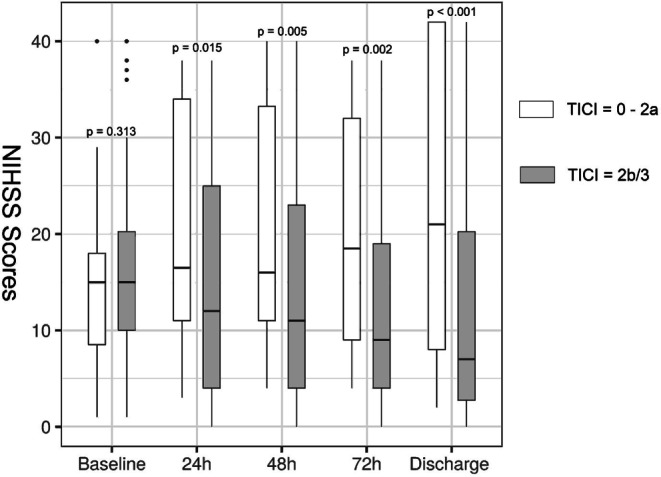
Boxplots showing treatment effect of endovascular thrombectomy on the change in National Institutes of Health Stroke Scale scores at 24-hour intervals and at discharge. Comparison of median values between groups was performed with Mann-Whitney U test. *NIHSS* National Institutes of Health Stroke Scale, *TICI* thrombolysis in cerebral infarction scale

172 out of 211 patients experienced successful reperfusion (TICI 2b/3) with 54 (31.4%) patients achieving ENI (Table 1). Of the remaining 39 patients with futile EVT (TICI 0–2a), only 5 patients were found to have ENI (12.8%). In addition, the mean NIHSS scores 24/48/72 h after EVT and particularly at discharge were significantly lower in patients with successful reperfusion (Fig. 2, Table 2).

**Table 2:** Daily change in National Institutes of Health Stroke Scale scores in patient groups with or without successful reperfusion

	Median (IQR) NIHSS score at admission	NIHSS score at 24 h	NIHSS score at 48 h	NIHSS score at 72 h	NIHSS score at discharge
*TICI 2b/3 (n* *=* *172)*	15 (10–20.75)	12 (4–25.25)	11 (4-23)	9 (4–19.25)	7 (2.25–20.75)
*TICI 0–2a (n* *=* *39)*	15 (8–18)	16.5 (11–34)	16 (11–33.75)	18.5 (8.75–34.25)	21 (7–42)
*p‑Values*	0.313	**0.015**	**0.005**	**0.002**	**<** **0.001**

Comparison of median values between groups was performed with Mann-Whitney U test. Significant *p*-values are marked in bold

*NIHSS* National Institutes of Health Stroke Scale, *TICI* thrombolysis in cerebral infarction scale

## Predictors of Early Neurological Improvement in Patients with Successful Reperfusion

In 105 patients with successful reperfusion as well as available CT perfusion imaging at baseline, favorable ENI was significantly associated with the time intervals from symptom onset to admission, groin puncture to reperfusion, age, mRS before stroke, baseline CTP ASPECTS and follow-up NCCT ASPECTS (Table 3).

**Table 3:** Predictors of early neurological improvement in patients with successful reperfusion

Variables	OR	CI (95%)	*p*-Values
Age (per year)	1.05	1.002–1.1	*0.042*
Sex	0.9	0.605–1.34	0.605
DM	0.997	0.68–1.46	0.987
CAD	0.982	0.67–1.442	0.928
Atrial fibrillation	0.702	0.468–1.053	0.087
Witnessed stroke	0.983	0.674–1.434	0.93
Symptom onset to admission (per 10 min)	0.978	0.966–0.99	*<* *0.001*
Door to groin (per 10 min)	1.003	0.887–1.135	0.96
Groin puncture to reperfusion (per 10 min)	0.731	0.631–0.845	*<* *0.001*
mRS before stroke (per point)	0.608	0.427–0.865	*0.006*
NIHSS at admission (per point)	0.977	0.908–1.051	0.533
Baseline CT ASPECTS (per point)	0.992	0.702–1.402	0.962
Baseline CTP ASPECTS (per point)	0.672	0.468–0.963	*0.03*
Volume of penumbra (per ml)	0.997	0.986–1.009	0.619
Volume of ischemic core (per ml)	0.97	0.939–1.003	0.076
Intravenous thrombolysis	0.968	0.656–1.428	0.87
First pass effect	0.896	0.629–1.276	0.542
Follow-up CT ASPECTS (per point)	1.749	1.322–2.314	*<* *0.001*
Hemorrhage on follow-up CT	0.934	0.656–1.332	0.708
SICH	1.074	0.862–1.34	0.523

Significant *p*-values are marked in *italic*

*OR* odds ratio, *CI* confidence interval, *DM* diabetes mellitus, *CAD* coronary artery disease, *mRS* modified Rankin scale, *NIHSS* National Institutes of Health Stroke Scale, *ASPECTS* Alberta Stroke Program early CT Score, *CTP* CT perfusion, *TICI* thrombolysis in cerebral infarction scale, *SICH* symptomatic intracranial hemorrhage

## Discussion

The main objective of this single-center retrospective analysis was to investigate treatment effect of EVT in patients due to anterior LVO and further to define predictors of favorable ENI in those with successful reperfusion. Short time intervals from symptom onset to hospital admission and from groin puncture to reperfusion, young patient age, absence of significant disability before stroke, and high follow-up ASPECTS score on NCCT after 24 h were significantly associated with favorable ENI. Baseline CTP ASPECTS outperformed baseline NCCT ASPECTS in predicting ENI; however, neither the volume of penumbra nor the size of ischemic core were associated with ENI.

First of all our study revealed again the paramount importance of achieving successful reperfusion (TICI 2b/3) for ischemic stroke outcome. In line with previous studies [15, 16], favorable ENI was more frequently present in patients with successful reperfusion (31.4% of patients with TICI 2b/3 versus 12.8% with TICI 0–2a). Moreover, the impact of successful reperfusion on neurological improvement grew daily until the day of discharge (Fig. 2), when the maximum difference of 14 points in median NIHSS scores between patients with and without successful reperfusion was reached.

In spite of advances in thrombectomy techniques and devices over the last decade, resulting in increasingly rapid and safe reperfusion of ischemic brain tissue, the reported percentage of patients with good functional outcomes (modified Rankin scale ≤ 2) at 90 days ranged from 24% to 51% [12, 13, 17–20]. Occlusion time from symptom onset to reperfusion has been proven as one of the most valid predictors for neurological outcomes [2, 16, 21, 22]. As demonstrated by the logistic regression model of our data, the time intervals from onset to hospital admission (OR 0.978 per 10 min, 95% CI 0.966–0.99, *p* < 0.001) and from groin puncture to reperfusion (OR 0.731 per 10 min, 95% CI 0.631–0.845, *p* < 0.001) were both critical determinants for ENI, but the intrahospital door-to-groin time was not. This might be explained by the standardized stroke workflow from diagnostic imaging at admission to preinterventional anesthesiological preparation in the angio suite. Nevertheless, ongoing efforts must be made to further shorten the intrahospital delay, not only to reduce the entire ischemic time but also to favor a higher rate of reperfusion. After all, this time interval was reported to be strongly associated with the final reperfusion grade and each additional hour led to a 26% reduction in the odds of TICI 2b/3 results [23]. Besides the time factor, our results also strengthened previous findings that high age and prethrombectomy disability of patients were associated with poor neurological improvement despite timely complete reperfusion [17, 24].

The ASPECTS score for evaluation of cerebral ischemic change on baseline NCCT is widely used to exclude patients with large infarct size from endovascular treatment due to less likelihood of functional independence and increased risk of hemorrhage [25, 26]. Baseline NCCT ASPECTS was proposed to be a strong predictor of neurological outcome in patients undergoing EVT due to anterior LVO with unknown or prolonged time window > 6 h [20]. The second analysis of the DAWN trial indicated as well that a higher ASPECTS was associated with greater benefit of EVT in patients presenting with a late window > 6 h [27]. In our study, the CTP ASPECTS turned out to be an independent predictor for favorable ENI (OR 0.672, 95%CI 0.468–0.963, *p* = 0.03), but not the baseline NCCT ASPECTS. It might be the short median time of approximately 3 h from onset to imaging that over 75% of the patients presented with ASPECTS of 9–10 at baseline. Indeed, baseline CT ASPECTS correlated inversely and tightly with the time from onset to hospital admission (ρ = −0.368, *p* = 0.002, supplementary material). This finding was supported by an analysis of 1329 ischemic stroke patients, in whom only 30% had an NCCT ASPECTS < 10 within the first 3 h after onset [6]. Thus, baseline CT ASPECTS appears to be more accurate and reproducible in patients presenting with a late time window > 6 h. On the contrary, the ischemic change of brain could be reliably assessed with CTP ASPECTS, which assessed the extent of ischemic core as areas with less than 30% CBF. Another advantage of CTP ASPECTS over NCCT ASPECTS is the increased diagnostic confidence and concordance of the readers in interpreting the imaging, as detection of early signs of cerebral ischemia on NCCT, such as subtle hypoattenuation or swelling, requires years of reading experience in acute stroke [5, 28]; however, additional radiation exposure and contrast agent administration as well as prolonged examination time by CTP imaging are known disadvantages in the workflow of stroke management. With the aid of artificial intelligence, accuracy of NCCT ASPECTS calculation might be improved in patients presenting in an early time window [29]. Consistent with previous work by SWIFT investigators [30], we found that higher ASPECTS on follow-up NCCT at 24 h was highly prognostic for ENI (OR 1.749 per point, 95%CI 1.322–2.314, *p* < 0.001).

In a meta-analysis by the HERMES collaboration with patients gaining more than 50% reperfusion following EVT [18], large ischemic core volume estimated by baseline perfusion imaging (CTP or MR diffusion imaging) was independently associated with worse functional outcome and each 10ml increase reduced the odds of favorable outcome by 20–30%. A second analysis of MR CLEAN trial and other previous work resulted in the same finding [31, 32]. Neither the volume of penumbra nor the size of ischemic core at baseline CTP were prognostic for favorable ENI in our study, though a large ischemic core volume tended to be associated with a reduced probability of ENI (OR 0.97, 95%CI 0.939–1.003, *p* = 0.076). The different definition of study endpoints (ENI versus mRS at day 90) might be one explanation. Another reason might be ischemic core overestimation on CTP, particularly in patients in earlier time windows and those with rapid reperfusion [33–35]. Due to potential presence of ghost infarct core on CTP, caution must be applied in decision-making for endovascular therapy in patients presenting with early time window within 3 h [36]. As the authors of the HERMES collaboration emphasized, the presence of a large ischemic core on baseline CTP should not prevent patients from the benefits of endovascular reperfusion because of the enhanced probability of functional independence with EVT compared to standard therapy alone at every level of ischemic core volume.

The main strengths of this study were its longitudinal documentation of NIHSS scores from admission to discharge with 24‑h intervals and availability of CT perfusion as a stroke imaging protocol, except for patients secondarily transferred from other hospitals. There were several limitations of our study. First of all, our results were based on retrospective analysis of data obtained in a single tertiary care center over the past 3 years. Residual measured and unmeasured confounding variables may have influenced some or all of the findings. It lacked cross-regional dissimilarity of baseline characteristics as well as a wider range of time windows. Second, compared to numerous well-known clinical trials [18, 23], the median age of our patients was significantly higher (76 years). This might mitigate the impact of endovascular treatment and other influencing factors on the early neurological outcomes. Third, 20% of patients with higher prestroke disability (mRS ≥ 3) were included in this study. The treatment effect of EVT might be further diluted. Last, we did not correlate the ENI with the final functional outcomes at 90 days, as this was not intended as a study subject given the many external validation trials.

## Conclusion

In patients with acute ischemic stroke due to anterior large vessel occlusion, achieving successful reperfusion (TICI 2b/3) is of paramount importance for early neurological improvement. Young age, absence of significant prestroke disability, short time intervals from onset to hospital admission and from groin puncture to reperfusion, high baseline CT perfusion ASPECTS and high non-contrast CT ASPECTS on follow-up CT at 24 h were associated with high frequency of early neurological improvement. Baseline CT perfusion ASPECTS outperformed baseline non-contrast ASPECTS in predicting early neurological improvement.

## References

[1] Goyal M, Menon BK, van Zwam WH, Dippel DW, Mitchell PJ, Demchuk AM, Dávalos A, Majoie CB, van der Lugt A, de Miquel MA, Donnan GA, Roos YB, Bonafe A, Jahan R, Diener HC, van den Berg LA, Levy EI, Berkhemer OA, Pereira VM, Rempel J, Millán M, Davis SM, Roy D, Thornton J, Román LS, Ribó M, Beumer D, Stouch B, Brown S, Campbell BC, van Oostenbrugge RJ, Saver JL, Hill MD, Jovin TG, HERMES collaborators (2016). Endovascular thrombectomy after large-vessel ischaemic stroke: a meta-analysis of individual patient data from five randomised trials. Lancet.

[2] Saver JL, Goyal M, van der Lugt A, Menon BK, Majoie CB, Dippel DW, Campbell BC, Nogueira RG, Demchuk AM, Tomasello A, Cardona P, Devlin TG, Frei DF, du Mesnil de Rochemont R, Berkhemer OA, Jovin TG, Siddiqui AH, van Zwam WH, Davis SM, Castaño C, Sapkota BL, Fransen PS, Molina C, van Oostenbrugge RJ, Chamorro Á, Lingsma H, Silver FL, Donnan GA, Shuaib A, Brown S, Stouch B, Mitchell PJ, Davalos A, Roos YB, Hill MD, HERMES Collaborators (2016). Time to Treatment With Endovascular Thrombectomy and Outcomes From Ischemic Stroke: A Meta-analysis. JAMA.

[3] Albers GW, Marks MP, Kemp S, Christensen S, Tsai JP, Ortega-Gutierrez S, McTaggart RA, Torbey MT, Kim-Tenser M, Leslie-Mazwi T, Sarraj A, Kasner SE, Ansari SA, Yeatts SD, Hamilton S, Mlynash M, Heit JJ, Zaharchuk G, Kim S, Carrozzella J, Palesch YY, Demchuk AM, Bammer R, Lavori PW, Broderick JP, Lansberg MG, DEFUSE 3 Investigators (2018). Thrombectomy for Stroke at 6 to 16 Hours with Selection by Perfusion Imaging. N Engl J Med.

[4] Nogueira RG, Jadhav AP, Haussen DC, Bonafe A, Budzik RF, Bhuva P, Yavagal DR, Ribo M, Cognard C, Hanel RA, Sila CA, Hassan AE, Millan M, Levy EI, Mitchell P, Chen M, English JD, Shah QA, Silver FL, Pereira VM, Mehta BP, Baxter BW, Abraham MG, Cardona P, Veznedaroglu E, Hellinger FR, Feng L, Kirmani JF, Lopes DK, Jankowitz BT, Frankel MR, Costalat V, Vora NA, Yoo AJ, Malik AM, Furlan AJ, Rubiera M, Aghaebrahim A, Olivot JM, Tekle WG, Shields R, Graves T, Lewis RJ, Smith WS, Liebeskind DS, Saver JL, Jovin TG, DAWN Trial Investigators (2018). Thrombectomy 6 to 24 Hours after Stroke with a Mismatch between Deficit and Infarct. N Engl J Med.

[5] Wang T, Chen L, Jin X, Yuan Y, Zhang Q, Shao C, Lu J (2021). CT perfusion based ASPECTS improves the diagnostic performance of early ischemic changes in large vessel occlusion. BMC Med Imaging.

[6] Gao J, Parsons MW, Kawano H, Levi CR, Evans TJ, Lin L, Bivard A (2017). Visibility of CT Early Ischemic Change Is Significantly Associated with Time from Stroke Onset to Baseline Scan beyond the First 3 Hours of Stroke Onset. J Stroke.

[7] Higashida RT, Furlan AJ, Roberts H, Tomsick T, Connors B, Barr J, Dillon W, Warach S, Broderick J, Tilley B, Sacks D, Technology Assessment Committee of the American Society of Interventional and Therapeutic Neuroradiology, Technology Assessment Committee of the Society of Interventional Radiology (2003). Trial design and reporting standards for intra-arterial cerebral thrombolysis for acute ischemic stroke. Stroke.

[8] Psychogios MN, Sporns PB, Ospel J, Katsanos AH, Kabiri R, Flottmann FA, Menon BK, Horn M, Liebeskind DS, Honda T, Ribo M, Ruiz MR, Kabbasch C, Lichtenstein T, Maurer CJ, Berlis A, Hellstern V, Henkes H, Möhlenbruch MA, Seker F, Ernst MS, Liman J, Tsivgoulis G, Brehm A (2021). Automated Perfusion Calculations vs. Visual Scoring of Collaterals and CBV-ASPECTS: Has the Machine Surpassed the Eye?. Clin Neuroradiol.

[9] von Kummer R, Broderick JP, Campbell BC, Demchuk A, Goyal M, Hill MD, Treurniet KM, Majoie CB, Marquering HA, Mazya MV, San Román L, Saver JL, Strbian D, Whiteley W, Hacke W (2015). The Heidelberg Bleeding Classification: Classification of Bleeding Events After Ischemic Stroke and Reperfusion Therapy. Stroke.

[10] Hacke W, Kaste M, Bluhmki E, Brozman M, Dávalos A, Guidetti D, Larrue V, Lees KR, Medeghri Z, Machnig T, Schneider D, von Kummer R, Wahlgren N, Toni D, ECASS Investigators (2008). Thrombolysis with alteplase 3 to 4.5 hours after acute ischemic stroke. N Engl J Med.

[11] Chalos V, van der Ende NAM, Lingsma HF, Mulder MJHL, Venema E, Dijkland SA, Berkhemer OA, Yoo AJ, Broderick JP, Palesch YY, Yeatts SD, Roos YBWEM, van Oostenbrugge RJ, van Zwam WH, Majoie CBLM, van der Lugt A, Roozenbeek B, Dippel DWJ, MR CLEAN Investigators (2020). National Institutes of Health Stroke Scale: An Alternative Primary Outcome Measure for Trials of Acute Treatment for Ischemic Stroke. Stroke.

[12] Heit JJ, Mlynash M, Kemp SM, Lansberg MG, Christensen S, Marks MP, Ortega-Gutierrez S, Albers GW (2019). Rapid Neurologic Improvement Predicts Favorable Outcome 90 Days After Thrombectomy in the DEFUSE 3 Study. Stroke.

[13] Wirtz MM, Hendrix P, Goren O, Beckett LA, Dicristina HR, Schirmer CM, Dalal S, Weiner G, Foreman PM, Zand R, Griessenauer CJ (2021). Predictor of 90-day functional outcome after mechanical thrombectomy for large vessel occlusion stroke: NIHSS score of 10 or less at 24 hours. J Neurosurg.

[14] Cilluffo G, Sottile G, La Grutta S, Muggeo VM (2020). The Induced Smoothed lasso: A practical framework for hypothesis testing in high dimensional regression. Stat Methods Med Res.

[15] Jayaraman MV, Grossberg JA, Meisel KM, Shaikhouni A, Silver B (2013). The clinical and radiographic importance of distinguishing partial from near-complete reperfusion following intra-arterial stroke therapy. AJNR Am J Neuroradiol.

[16] Mazighi M, Meseguer E, Labreuche J, Serfaty JM, Laissy JP, Lavallée PC, Cabrejo L, Guidoux C, Lapergue B, Klein IF, Olivot JM, Rouchaud A, Desilles JP, Schouman-Claeys E, Amarenco P (2012). Dramatic recovery in acute ischemic stroke is associated with arterial recanalization grade and speed. Stroke.

[17] van Horn N, Kniep H, Leischner H, McDonough R, Deb-Chatterji M, Broocks G, Thomalla G, Brekenfeld C, Fiehler J, Hanning U, Flottmann F (2021). Predictors of poor clinical outcome despite complete reperfusion in acute ischemic stroke patients. J Neurointerv Surg.

[18] Campbell BCV, Majoie CBLM, Albers GW, Menon BK, Yassi N, Sharma G, van Zwam WH, van Oostenbrugge RJ, Demchuk AM, Guillemin F, White P, Dávalos A, van der Lugt A, Butcher KS, Cherifi A, Marquering HA, Cloud G, Macho Fernández JM, Madigan J, Oppenheim C, Donnan GA, Roos YBWEM, Shankar J, Lingsma H, Bonafé A, Raoult H, Hernández-Pérez M, Bharatha A, Jahan R, Jansen O, Richard S, Levy EI, Berkhemer OA, Soudant M, Aja L, Davis SM, Krings T, Tisserand M, San Román L, Tomasello A, Beumer D, Brown S, Liebeskind DS, Bracard S, Muir KW, Dippel DWJ, Goyal M, Saver JL, Jovin TG, Hill MD, Mitchell PJ, HERMES collaborators (2019). Penumbral imaging and functional outcome in patients with anterior circulation ischaemic stroke treated with endovascular thrombectomy versus medical therapy: a meta-analysis of individual patient-level data. Lancet Neurol.

[19] Seker F, Fiehler J, Möhlenbruch MA, Herweh C, Flottmann F, Ringleb PA, Thomalla G, Steiner T, Kraemer C, Brekenfeld C, Bendszus M (2021). Clinical Outcome After Endovascular Thrombectomy in 3 Triage Concepts: A Prospective, Observational Study (NEUROSQUAD). Stroke.

[20] Nagel S, Herweh C, Pfaff JAR, Schieber S, Schönenberger S, Möhlenbruch MA, Bendszus M, Ringleb PA (2019). Simplified selection criteria for patients with longer or unknown time to treatment predict good outcome after mechanical thrombectomy. J Neurointerv Surg.

[21] Prabhakaran S, Castonguay AC, Gupta R, Sun CJ, Martin CO, Holloway W, Mueller-Kronast NH, English J, Linfante I, Dabus G, Malisch T, Marden F, Bozorgchami H, Xavier A, Rai A, Froehler M, Badruddin A, Taqi MA, Novakovic R, Abraham M, Janardhan V, Shaltoni H, Yoo AJ, Abou-Chebl A, Chen P, Britz G, Kaushal R, Nanda A, Nogueira R, Nguyen T, Zaidat OO (2017). Complete reperfusion mitigates influence of treatment time on outcomes after acute stroke. J Neurointerv Surg.

[22] Prothmann S, Schwaiger BJ, Gersing AS, Reith W, Niederstadt T, Felber A, Kurre W (2017). Acute Recanalization of Thrombo-Embolic Ischemic Stroke with pREset (ARTESp): the impact of occlusion time on clinical outcome of directly admitted and transferred patients. J Neurointerv Surg.

[23] Bourcier R, Goyal M, Liebeskind DS, Muir KW, Desal H, Siddiqui AH, Dippel DWJ, Majoie CB, van Zwam WH, Jovin TG, Levy EI, Mitchell PJ, Berkhemer OA, Davis SM, Derraz I, Donnan GA, Demchuk AM, van Oostenbrugge RJ, Kelly M, Roos YB, Jahan R, van der Lugt A, Sprengers M, Velasco S, Lycklama À, Nijeholt GJ, Hassen BW, Burns P, Brown S, Chabert E, Krings T, Choe H, Weimar C, Campbell BCV, Ford GA, Ribo M, White P, Cloud GC, San Roman L, Davalos A, Naggara O, Hill MD, Bracard S, HERMES Trialists Collaboration (2019). Association of Time From Stroke Onset to Groin Puncture With Quality of Reperfusion After Mechanical Thrombectomy: A Meta-analysis of Individual Patient Data From 7 Randomized Clinical Trials. JAMA Neurol.

[24] Weyland CS, Mokli Y, Vey JA, Kieser M, Herweh C, Schönenberger S, Bendszus M, Möhlenbruch MA, Ringleb PA, Nagel S (2021). Predictors for Failure of Early Neurological Improvement After Successful Thrombectomy in the Anterior Circulation. Stroke.

[25] Yoo AJ, Zaidat OO, Chaudhry ZA, Berkhemer OA, González RG, Goyal M, Demchuk AM, Menon BK, Mualem E, Ueda D, Buell H, Sit SP, Bose A, Penumbra Pivotal and Penumbra Imaging Collaborative Study (PICS) Investigators (2014). Impact of pretreatment noncontrast CT Alberta Stroke Program Early CT Score on clinical outcome after intra-arterial stroke therapy. Stroke.

[26] Jovin TG, Chamorro A, Cobo E, de Miquel MA, Molina CA, Rovira A, San Román L, Serena J, Abilleira S, Ribó M, Millán M, Urra X, Cardona P, López-Cancio E, Tomasello A, Castaño C, Blasco J, Aja L, Dorado L, Quesada H, Rubiera M, Hernandez-Pérez M, Goyal M, Demchuk AM, von Kummer R, Gallofré M, Dávalos A, REVASCAT Trial Investigators (2015). Thrombectomy within 8 hours after symptom onset in ischemic stroke. N Engl J Med.

[27] Bhuva P, Yoo AJ, Jadhav AP, Jovin TG, Haussen DC, Bonafe A, Budzik RJ, Yavagal DR, Hanel RA, Hassan AE, Ribo M, Cognard C, Sila CA, Morgan PM, Zhang Y, Shields R, Smith W, Saver JL, Liebeskind DS, Nogueira RG, DAWN Trial Investigators (2019). Noncontrast Computed Tomography Alberta Stroke Program Early CT Score May Modify Intra-Arterial Treatment Effect in DAWN. Stroke.

[28] Wardlaw JM, Farrall AJ, Perry D, von Kummer R, Mielke O, Moulin T, Ciccone A, Hill M, Acute Cerebral CT Evaluation of Stroke Study (ACCESS) Study Group (2007). Factors influencing the detection of early CT signs of cerebral ischemia: an internet-based, international multiobserver study. Stroke.

[29] Zeleňák K, Krajina A, Meyer L, Fiehler J, Esmint Artificial Intelligence And Robotics Ad Hoc Committee (2021). How to Improve the Management of Acute Ischemic Stroke by Modern Technologies, Artificial Intelligence, and New Treatment Methods. Life (Basel).

[30] Liebeskind DS, Jahan R, Nogueira RG, Jovin TG, Lutsep HL, Saver JL, SWIFT Investigators (2014). Serial Alberta Stroke Program early CT score from baseline to 24 hours in Solitaire Flow Restoration with the Intention for Thrombectomy study: a novel surrogate end point for revascularization in acute stroke. Stroke.

[31] Borst J, Berkhemer OA, Roos YB, van Bavel E, van Zwam WH, van Oostenbrugge RJ, van Walderveen MA, Lingsma HF, van der Lugt A, Dippel DW, Yoo AJ, Marquering HA, Majoie CB, MR CLEAN investigators (2015). Value of Computed Tomographic Perfusion-Based Patient Selection for Intra-Arterial Acute Ischemic Stroke Treatment. Stroke.

[32] Sanák D, Nosál’ V, Horák D, Bártková A, Zelenák K, Herzig R, Bucil J, Skoloudík D, Burval S, Cisariková V, Vlachová I, Köcher M, Zapletalová J, Kurca E, Kanovský P (2006). Impact of diffusion-weighted MRI-measured initial cerebral infarction volume on clinical outcome in acute stroke patients with middle cerebral artery occlusion treated by thrombolysis. Neuroradiology.

[33] García-Tornel Á, Campos D, Rubiera M, Boned S, Olivé-Gadea M, Requena M, Ciolli L, Muchada M, Pagola J, Rodriguez-Luna D, Deck M, Juega J, Rodríguez-Villatoro N, Sanjuan E, Tomasello A, Piñana C, Hernández D, Álvarez-Sabin J, Molina CA, Ribó M (2021). Ischemic Core Overestimation on Computed Tomography Perfusion. Stroke.

[34] Bivard A, Kleinig T, Miteff F, Butcher K, Lin L, Levi C, Parsons M (2017). Ischemic core thresholds change with time to reperfusion: A case control study. Ann Neurol.

[35] Tsang ACO, Lenck S, Hilditch C, Nicholson P, Brinjikji W, Krings T, Pereira VM, Silver FL, Schaafsma JD (2020). Automated CT Perfusion Imaging Versus Non-contrast CT for Ischemic Core Assessment in Large Vessel Occlusion. Clin Neuroradiol.

[36] Boned S, Padroni M, Rubiera M, Tomasello A, Coscojuela P, Romero N, Muchada M, Rodríguez-Luna D, Flores A, Rodríguez N, Juega J, Pagola J, Alvarez-Sabin J, Molina CA, Ribó M (2017). Admission CT perfusion may overestimate initial infarct core: the ghost infarct core concept. J Neurointerv Surg.

